# The KT Jeang retrovirology prize 2024: Walther Mothes

**DOI:** 10.1186/s12977-024-00649-8

**Published:** 2024-10-24

**Authors:** Walther Mothes

**Affiliations:** Yale University, New Haven, USA


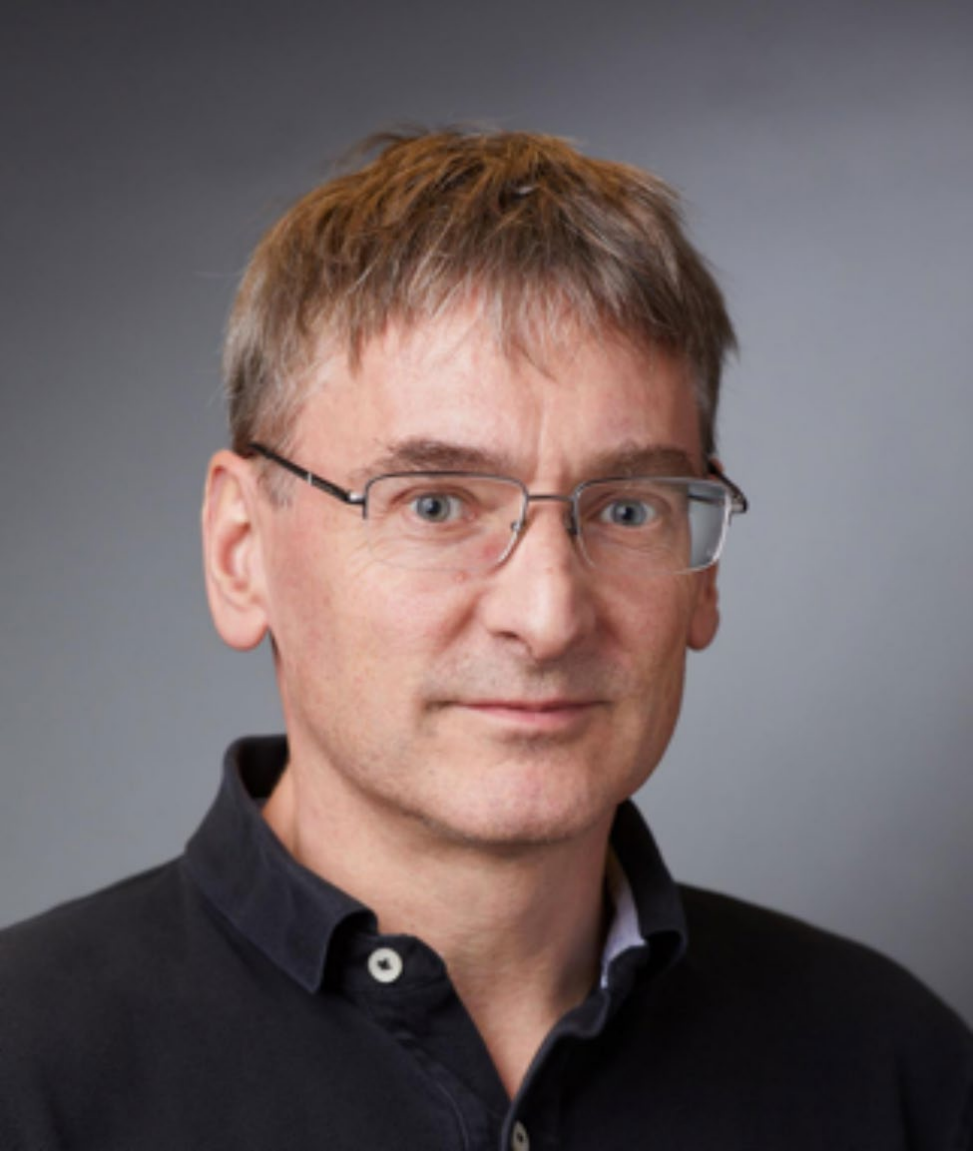


Walther Mothes studied chemistry at the Friedrich Schiller University Jena and Humboldt University, Berlin, Germany. While studying, his interests shifted from organic chemistry to biochemistry and cell biology. Beginning in 1992, Walther was fortunate to work part-time in Tom Rapoport’s lab at the Max Delbrück Center in Berlin, Germany, where he studied protein secretion at the endoplasmic reticulum (ER). Tom’s lab was unusual as it had an American-style atmosphere where all members, students or professors, were addressed with an informal “you”. For Walther, this was a welcome departure and offered a sense of liberation within the prevailing hierarchical East German academic culture. Most importantly, Tom’s lab offered a scientifically exciting environment to draw talented young scientists to his lab back then and continues to do so today. On the opposite bench, Dirk Gorlich identified TRAM and Sec61, and subsequently reconstituted protein translocation from purified components [1–3]. Walther’s project was to probe the environment of a polypeptide chain as it emerges from the ribosome and crosses through the ER using site-specific photo-crosslinking. His investigations demonstrated that Sec61 is in vicinity of the emerging polypeptide chain at a distance from the ribosome where amino acids were expected to be in the membrane. These data provided early evidence that Sec61 forms a channel for secretory proteins [4]. Walther subsequently joined the Rapoport lab for his PhD thesis work and moved with the lab to Harvard Medical School, Boston. There, he investigated the process by which membrane proteins are incorporated into the endoplasmic reticulum. Using an initial version of Amber suppression technology to site specifically incorporate photo-crosslinkers, Walther showed that the Sec61 channel can laterally open to release membrane anchors into the lipid bilayer (1993, Ph.D. in Cell Biology from Humboldt University, Berlin, Germany) [5, 6].

For his postdoctoral training, Walther joined John Young’s lab across the Harvard Medical School quad at the time, to start working on retroviruses. John had an incredibly uplifting and positive personality, and the lab had a vibrant atmosphere, primarily populated by graduate students. Walther studied the entry pathway of the avian leukosis virus (ALV), which at the time was considered a model virus for an HIV-1 like receptor-activated and pH-independent entry mechanism. However, when Walther used more direct PCR-based assays that detect early RT products to measure entry events, he discovered that although ALV primed its viral glycoprotein by binding to the receptor, it still relied on a low pH-dependent endocytic entry pathway [7]. Walther’s postdoctoral training in the Young lab was not without challenges as just three months in, John Young announced his move to Madison, Wisconsin. Walther sought other options as he was unable to leave Boston because his girlfriend, later his wife, Tilla, had recently matched into residency at Tufts University. Fortunately, Jim Cunningham agreed to host Walther in his lab where he developed tools to study entry of murine leukemia viruses (MLV). During these few years Walther also collaborated with Ute Felbor in the Olsen laboratory on tumor angiogenesis [8, 9].

Walther Mothes started his own laboratory in 2001 at the Yale School of Medicine in New Haven, Connecticut. Realizing his proficiency in applying spatio-visual approaches to biological questions, he became interested in directly visualizing various steps of the retroviral life cycle. Fluorescently labeled MLV Gag (Gag-GFPs) allowed insights into virus budding and release, while fluorescently labeled Env protein allowed monitoring of how MLV enters the cell [10, 11]. Using these tools, Maik Lehmann, first post-doctoral fellow in the lab, discovered the ability of retroviruses to “surf” along filopodia of cells to reach perinuclear areas more permissive for cell entry, a movement driven by underlying retrograde F-actin flow [11]. Nathan Sherer, first graduate student in the lab, focused on visualizing retrovirus budding and discovered that MLV particles often remained associated with the surface of cells [12]. He realized that retainment at the surface of producer cells, followed by virus surfing on target cells, could result in cell-to-cell transmission of viruses if infected cells and non-infected cells were co-cultured. These experiments resulted in the early documentation of virus cell-to-cell transmission via filopodial bridges [13, 14]. Jing Jin then applied 4D imaging to capture de novo assembly sites and observed that MLV assembly is polarized towards sites of cell-cell contact [15].

To what extent studies on retroviral spread in tissue culture cells were relevant for the spread of viruses in vivo was unknown. To address this question, Xaver Sewald established in vivo imaging of virus dissemination in living animals using multiphoton microscopy [16]. Xaver found that MLV was captured by subcapsular sinus or metallophillic macrophages. Retrovirus capture was mediated by an interaction between gangliosides on virus particles and Siglec-1/CD169 expressed on macrophages [16]. MLV-laden macrophages then formed long-lived synaptic contacts to trans-infect permissive B cell subsets. Infected B cells subsequently migrated to the inter-follicular space to spread the infection to susceptible lymphocytes through virological synapses [16, 17]. Efficient infection of mice required CD169, suggesting that retroviruses utilize a combination of fluid-based movement followed by CD169-dependent trans-infection of permissive lymphocytes to spread in vivo. This work demonstrated that concepts, developed entirely in vitro to explain efficient retroviral spread, such as trans-infection and virological synapses, exist in vivo [16].

While studying murine retroviruses had allowed the lab to gain fundamental insights into virus assembly, transmission, and entry into cells, Peng Zhong and Luis Agosto did the heavy lifting to establish HIV-1 research in the Mothes lab [18–20]. Their studies on HIV-1 cell-to-cell transmission confirmed earlier findings by other labs that it is significantly more efficient than cell-free spread, overcoming multiple barriers including restriction factors and reducing antiretroviral therapy (ART) efficacy due to high viral multiplicity of infection (MOI) at cell-cell contact sites [18–20]. While some inhibitors were less effective, combination therapy remained potent [20]. This study revealed the importance of targeting high MOI while designing ART regimens and highlighted cell-to-cell transmission as a critical factor in ART success and antiviral therapy design.

Virus dissemination studies broadened the focus of the lab towards more immunological questions as virus spread in an animal is intricately intertwined with innate and adaptive immune responses. Pradeep Uchil, who introduced an interest in viral restriction factors such as members of the TRIM E3 ligase family proteins [21–23], realized with Ruoxi Pi, a graduate student, that CD169-mediated capture and trans-infection of permissive B-cells required innate sensing of retroviruses to permit virus amplification and subsequent spread [24]. Importantly, while retroviruses native to mice, evolved to exploit CD169-mediated infection for persistence, CD169-orchesterated protective immune responses were dominant against highly pathogenic retroviruses and promoted virus clearance [25].

Pradeep Uchil, working with Kelsey Haugh and John Ventura, then developed bioluminescence imaging to monitor retrovirus infection in the same animal over time, as well as oral transmission of virus from dam to pups [16, 26–29]. During the pandemic, when Walther like all PIs at Yale had to work from home, Pradeep Uchil was one of the senior scientists in the lab leading the applications of the lab’s technologies to COVID-19 research. Pradeep working together with Irfan Ullah from Priti Kumar’s lab, established bioluminescence imaging for SARS-CoV-2 to demonstrate that Fc-effector functions of antibodies played an important role in the ability of antibodies to protect animals against SARS-CoV-2-induced mortality [29]. Fc effector functions also play an important role in protecting against emerging variants of concerns that escape existing antibodies because non-neutralizing antibodies with Fc effector functions tend to recognize variant-agnostic epitopes on the viral spike protein [30]. Bioluminescence imaging of SARS-CoV-2 dissemination greatly facilitated studies on antibody-mediated immunity against SARS-CoV-2, vaccine design as well as other therapeutic interventions [31–33]. As such, the lab founded with an interest in live-cell imaging by fluorescently labeling viral particles, over the years developed the tools that permit imaging of virus dissemination and pathogenesis at various scales of resolution, from whole animals down to monitoring single cells at the tissue level [28]. Moreover, the long-standing interest of the lab in virus dissemination was complemented by the question of how antibody-mediated immunity interferes with viral spread.

A new direction of research was initiated when James Munro, a biophysicist by training, introduced single molecule Förster resonance energy transfer (smFRET) imaging into the lab. Originally being interested in learning virology, James and Walther realized that smFRET imaging can be very powerful to reveal conformational dynamics of viral glycoproteins on the surface of virus particles. James’ smFRET studies revealed that the unliganded HIV-1 Env protein is intrinsically dynamic, and that activation by the receptor CD4 stabilizes a pre-existing conformational state through a necessary structural intermediate [34]. Elegant smFRET experiments by Xiaochu Ma indicated that this intermediate likely corresponds to an asymmetric trimer formed when a single CD4 initially engages the trimer [35]. smFRET also allowed the characterization of the conformational preferences of neutralizing antibodies and to inform immunogen design [34, 36–40]. Maolin Lu then compared the smFRET states of Env on the surface of the virus with that of commonly used soluble trimers and made the unexpected observation that a predominant conformational state on the virus had not yet been structurally characterized [39]. To resolve these open questions, it became necessary to flank the smFRET approach with an orthogonal structural method such as cryo-electron tomography that similarly allowed structural characterization of Env on the surface of viruses. This became possible thanks to a collaboration with Jun Liu and the transition of Wenwei Li from the Liu lab to the Mothes lab [41]. Wenwei Li expanded cryoET methods to characterize Env-receptor interactions in biological membranes [42]. Wenwei observed the HIV-1 Env trimer engaged only a single CD4 receptor molecule when membranes were further apart, binding of a second and a third CD4 receptor molecule as membranes were closer together [42]. Importantly, the Env trimers bound to one and two CD4 molecules were clearly asymmetric as Xiaochu Ma’s smFRET experiments had predicted earlier [35]. Parallel work by the Bjorkman lab determined the structures of asymmetric HIV Env trimers by single particle EM, providing atomic models for these asymmetric trimers that agreed well with cryoET density maps [43]. While open questions remain, these findings raise the hope that a combined cryoET, single particle EM and smFRET approach, supported by computational methods, can arrive at a comprehensive understanding of the structure and dynamics of viral spike proteins.

Just like certain Env conformations are vulnerable to antibodies, they are also vulnerable to cell-intrinsic restriction factors such as the SERINC family of proteins. Studying the underlying mechanism in collaboration with the Yeager lab, Jonathan Grover and Ziwei Yang discovered that SERINC proteins are lipid scramblases which impact membrane asymmetry. This lipid scramblase activity correlated with changes in Env conformation and loss of infectivity [44]. Accordingly, a cellular scramblase TMEM16F, sent into virus particles, scrambled lipids and affected virus infectivity [44].

When the COVID-19 pandemic hit, Maolin Lu, Jonathan Grover and Wenwei Li applied these single molecule technologies developed to study HIV-1 Env to understand the structure and dynamics of the SARS-CoV-2 spike protein and characterize how it is recognized by antibodies [45, 46]. Michael Grunst became interested in visualizing how SARS-CoV-2 spike mediates membrane fusion. Towards this end, he studied spike-ACE2 receptor interactions and activation for fusion in membranes [47]. ACE2 receptor dimers were found to crosslink spike proteins and the prehairpin intermediate was a distinct intermediate during membrane fusion. Intriguingly, anti-stem helix antibodies bound to the prehairpin intermediate and arrested spike refolding thereby preventing membrane fusion [47]. Given that the fusion machine is conserved across beta-coronaviruses, identifying such vulnerabilities during spike refolding can inform the design of pan-sarbecovirus vaccines. Encouraged by these studies on coronaviruses, the Mothes lab is currently similarly studying how refolding of HIV-1 gp41 drives membrane fusion.

In addition to single molecule studies, the lab continues to have an interest in virus dissemination, cell-to-cell transmission, antiviral immunity and studies these processes across spatial and temporal resolutions. Research in the Mothes lab is a joint journey with Pradeep Uchil and would not have been possible without the many talented and enthusiastic summer students, under graduates, graduate students and postdocs who have worked in the lab. The Mothes lab has also greatly benefited from the generous support from many colleagues including Peter Kwong, Andres Finzi, Priti Kumar, Scott Blanchard, Joseph Sodroski, James Munro, Mark Ladinsky and Pamela Bjorkman to name a few.

Walther Mothes is an advocate for trainees and served as the director of graduate studies for the program in microbiology at Yale for 9 years. He is on the editorial board of the Journal of Virology, co-organized the Cold Spring Harbor Retroviruses Meeting in 2018, and is a member of the NIH study section “HIV Molecular Virology, Cell Biology and Drug Development” (HVCD). Walther is proud of his NIH MERIT Award by the Director and Staff of the Division of AIDS, NIAID (2023). When not working, Walther enjoys hiking with his wife along the New England Trail in Connecticut, in the Appalachian Mountains, Adirondacks and Rocky Mountains.
